# Immature and mature bone marrow-derived dendritic cells exhibit distinct intracellular mechanical properties

**DOI:** 10.1038/s41598-023-28625-w

**Published:** 2023-02-03

**Authors:** Antoine Leblanc-Hotte, Cindy Audiger, Geneviève Chabot-Roy, Félix Lombard-Vadnais, Jean-Sébastien Delisle, Yves-Alain Peter, Sylvie Lesage

**Affiliations:** 1 Immunology-Oncology Research Axis, Maisonneuve-Rosemont Hospital Research Centre, Montreal, QC Canada; 2 Département de Microbiologie, Infectiologie et Immunologie, Université de Montréal, Montreal, QC Canada; 3 Department of Engineering Physics, Polytechnique Montréal, Montreal, QC Canada; 4 Département de Médecine, Université de Montréal, Montreal, QC Canada

**Keywords:** Biophysics, Biotechnology, Immunology, Biological techniques, Lab-on-a-chip, Cell biology, Cell migration

## Abstract

Dendritic cells (DCs) patrol the organism at an immature stage to detect the presence of pathogens. Once activated, these mature DCs reach the lymph nodes to activate antigen-specific T lymphocytes and thus initiate an adaptative immune response to control the pathogen. The migration of both immature and mature DCs is a key process for their optimal function. DC migration requires transit through narrow constrictions that is allowed by their high local and global deformation capabilities. In addition to cytoplasmic changes, the nucleus mechanical properties also have a major impact for cellular migration and motility. Yet, nucleus intracellular mobility of dendritic cells or its variation upon maturation have not been investigated. Our study defines the biophysical phenotypic variations of dendritic cells upon maturation using interferometric deformability cytometry. This method characterizes different cellular mechanical properties, such as elongation and nucleus offset, by assessing the refractive index spatial distribution of shear-induced deformed cells. By using these parameters, our data suggest that in vitro bone marrow derived dendritic cell (BMDC) maturation induces cell stiffening and reduces nucleus mobility, allowing to distinguish immature and mature dendritic cells. Overall, our method provides insights on intracellular mechanical properties of two dendritic cell states.

## Introduction

Dendritic cells (DCs) play a crucial role in initiating adaptive immune responses^1^. At their immature state, they are present in both lymphoid and non-lymphoid tissues, where they continuously sample their environment to detect the presence of pathogens. This will induce their maturation, migration, and the presentation of pathogen-derived peptides to T cells in the lymph nodes^2^. Thus, immature, and mature DCs present with different roles in the initiation of adaptive responses, which are associated with specific features in terms of phenotype, function, and mechanical properties.

Immature DCs are relatively immobile and have a stellate morphology with dendrites. They use these extensions to undergo sweeping movements to screen the environment and capture antigens inside the tissue or organs through phagocytosis or endocytosis^3^. Immature DCs thus require the ability to rearrange their cytoskeleton to allow the engulfment of antigens. However, as soon as they mature, DCs reduce their phagocytosis ability, increase their motility and leave the tissue. They cross the endothelium of lymphatic vessels and migrate to the draining lymph nodes. These mature DCs will then interact with T cells to initiate an adaptive response^3^. Overall, immature and mature DCs are exposed to distinct environments. Whereas immature DCs continually sample their environment inside the tissue, mature DCs need to migrate through the lymphatic vessels and make strong interactions with T cells in the lymph nodes.

Mature DCs have a higher cortical stiffness than immature DCs, a characteristic that is conserved in murine splenic DCs, murine bone marrow-derived DCs (BMDCs)^4–6^ and human monocyte-derived DCs^7, 8^. This increase in cortical stiffness is dependent on the DCs capacity to reprogram their cytoskeleton. Indeed, treatment of BMDCs with actin-depolymerizing agents does not change the stiffness of immature DCs but reduces the stiffness of mature DCs^9^. This reduction in stiffness of mature DCs diminishes their ability to activate T cells in vitro, emphasizing the close link between maturation, cytoskeleton, cellular stiffness, and immune function. In addition, in human monocyte-derived DCs, the higher stiffness induced by maturation is associated with a change in cytoplasmic lipid composition, which increases the fluidity and polarity of the membrane^10^.

Another important parameter limiting the deformability of cells is the nucleus^11^. As a cell changes in morphology or elongates, it requires the ability to deform the nucleus in a manner that parallels the shape of the cell. However, the nucleus intracellular mobility of immature and mature DCs has yet to be reported.

We have previously developed a vanguard microphotonic method named interferometric deformability cytometry^12^. This method consists of an optically resonant Fabry-Pérot microcavity highly sensitive to the refractive index (RI). Cells are driven through the cavity in a rectangular microchannel inducing cellular deformation by shear stress due to the proximity to the walls. Shear-induced deformability cytometry, such as real-time (RT)-deformability cytometry, is considered more sensitive to cellular structures at or near the cell surface, such as the cell membrane and the actin network, because of the smaller stress amplitude and strain rate^13^. Conversely, extensional deformability cytometry, which generates large compressive fluidic stresses at large strain rates, is considered more sensitive to deeper cellular structures, such as the nucleus. RT-deformability cytometry and extensional deformability are both fast imaging techniques in which deformed cell profiles are captured. Computed deformation-related indices provide valuable information about mechanical phenotypes of assessed cells. In RT-deformability cytometry, cells flow along a square microchannel in which shear stress induces deformation. In extensional deformability cytometry, cells cross a microchannel intersection, with opposing flow directions, which creates great fluidic stress and thus large cellular deformation. The main advantages of shear-induced deformation by interferometric deformability cytometry are that it does not require imaging and that it can provide relevant optical and intracellular mechanical phenotyping.

Using interferometric deformability cytometry, we provide further insights to the phenotypic variations of mouse BMDCs upon maturation with lipopolysaccharide (LPS). Our study reveals that our shear-induced interferometric deformability cytometry method is sensitive to deep cellular structures (namely, the nucleus), even at small shear stresses. By assessing the RI spatial distribution, interferometric deformability cytometry can perceive intracellular organization without requiring image recording or fluorescence. We report two distinct nuclei localization states in immature BMDCs, which are correlated to the fluidic stress amplitude. In addition, we exploit the fact that maturation of BMDCs induces whole cell stiffening and, as such, reduces nucleus mobility that maturation of BMDCs induced whole cell stiffening and reduced nucleus mobility. Markedly, interferometric deformability cytometry provides the sensitivity and throughput required for explicit discrimination between immature and mature BMDCs states. To our knowledge, this is the first time that nucleus localization and mobility are used to characterize large populations of DCs and discriminate immature from mature BMDCs. This highlights the specific mechanical properties associated with the maturation stage of DCs.

## Materials and methods

### Device fabrication

Fabrication of interferometric deformability cytometry devices is detailed in previous publications^12, 14^. Briefly, microphotonic and microfluidic structures were defined on a silicon-on-insulator wafer using photolithography and deep reactive ion etching. The two distributed Bragg reflectors composing the Fabry-Pérot cavity were designed to achieve resonances in the near infrared region, at ~ 1550 nm. Vertical thin layers of silicon alternated with air composing the distributed Bragg reflectors are ~ 1.5 μm. Rib waveguides coupling light from optical fibers to the microcavity were designed to yield a single mode-like propagation^15^. Small aspect ratio $AR=H/W<1$ rectangular microchannels were designed to provide sheathless inertial focusing of the cells as well as longitudinal spacing. For all fabricated devices, the microchannel height was set at 15 μm by the thickness of the silicon-on-insulator top silicon layer. Microchannel widths ranged from 30 to 50 μm. Devices were diced using a standard diamond blade technique, sealed with anodically bonded Pyrex plates and placed in a 3D printed clamping platform for robust tubing connections.

### Mice

C57BL/6 (B6, #000664) mice were purchased from Jackson Laboratory (Bar Harbor, United States). Both male and female mice were used. All mice used for experiments were aged between 7 and 11 weeks. All mice were maintained at the Maisonneuve-Rosemont Hospital animal facility. The Maisonneuve-Rosemont Hospital ethics committee, overseen by the Canadian Council for Animal Protection, approved the experimental procedures. All methods were carried out in accordance with relevant guidelines and regulations.

### BMDCs

Bone marrow was harvested from femurs, tibias, and iliac crests of B6 mice. Red blood cells were lysed in a NH_4_Cl solution, and the remaining cells were collected following centrifugation. Bone marrow cells were resuspended at a concentration of 1.5 × 10^6^ cells/mL in complete RPMI containing 100 ng/ml of FLT3L (Bio X Cell, Lebanon, NH) and incubated at 37 °C with 5% CO_2_ over 7 days, following standard protocol for BMDCs differentiation^16^. At day 6, cells were stimulated (mature BMDCs) or not (immature BMDCs) with 1 μg/ml LPS (Sigma, Saint-Louis, MO) overnight to induce the BMDC maturation. At day 7, immature and mature BMDCs were collected and analyzed by both flow cytometry and interferometric deformability cytometry.

### Flow cytometry

Cell suspensions were labeled with antibodies from BioLegend (San Diego, CA) listed as antigen (clone): CD11c (N418), IA/IE (M5/114.15.2), CD40 (1C10), CD80 (16-10A1), CD86 (GL1), and Zombie Aqua Viability Dye. All samples were acquired on a Fortessa (BD Biosciences, Franklin Lakes, NJ) and were analyzed with the FlowJo software (FlowJo, BD Biosciences). For all flow cytometry analyses, doublets were excluded using forward and side scatter width versus height and dead cells were excluded based on Zombie Aqua Viability Dye. DCs were define as CD11c^+^. Efficient maturation of the BMDC following LPS stimulation was assessed for each experiment, by flow cytometry, based on the upregulation of CD40, CD80, CD86 and I-A expression levels in comparison to immature DCs.

### Interferometric deformability cytometry

Operation of interferometric deformability cytometry devices is detailed in previous publications^12, 14^. Cells injected by a syringe pump (Harvard Apparatus, Pump 11 Pico Plus Elite) shift the optical resonances of the Fabry–Perot interferometer towards longer wavelengths when flowing through its cavity. A tunable laser source (Agilent, 8164B) locked the emitted wavelength to match the resonance peak in absence of cells. Specific wavelengths used for all reported data of this paper range between 1550 and 1600 nm. A fast InGaAs infrared photodetector (Thorlabs, DET01CFC) recorded the optical power variations in time and relayed it to a high-speed digitizer (National Instruments, NI-PXI 5114). A custom MatLab algorithm as well as FlowJo were used to perform curve computation and population analysis respectively. A sensitivity of approximately 950 nm/RIU was previously measured using certified refractive index oils, thus corresponding to a limit of detection of $1.6\times {10}^{-5} \mathrm{RIU}$ when considering an experimentally measured accuracy of $3\sigma =0.015 \mathrm{nm}$^12^. Debris and dead cells were excluded based on their smaller *maximum* and *cell time* values whereas doublets and artefacts were excluded based on the larger curve width.

### Numerical simulations

Shear stress, pressure, settling velocity and deformation profile of a model cell flowing through a small aspect ratio AR = H/W < 1 rectangular channel were computed using finite element method simulations (COMSOL Multiphysics software) similar to the one reported in Mietke et al., 2015. First, the settling velocities were determined by the forces equilibrium between pressure and shear stress, in the flowing direction, on a rigid advecting sphere. To do so, a fully developed flow rate was launched at the rectangular microchannel inlet and interacted with hollow spherical rigid boundaries, placed in the center of the microchannel. These spherical boundaries were given a fixed velocity in the direction of the flow. All boundaries had the no slip conditions, except for the inlet and outlet, and stationary laminar flow equations were solved. The outlet was kept at a static null pressure. Total stress in the flowing direction was assessed and spherical rigid boundaries velocity was incremented until the total stress value was below 1 × 10^–4^ μN. These settling velocities were computed for flow rates ranging from 5 to 135 μl/min. Then, the deformation of a homogeneous sphere was computed at the settling velocities. Specifically, the hollow spherical rigid boundaries of the previous simulation phase were replaced by a filled sphere of isotropic linear elastic material (E = 2 kPa, ν = 0.49 and ρ = 1100 kg/m^3^) for which the settling velocity was imposed on the outer boundaries. The filled sphere center was constrained to a fixed position to avoid induced advection. All other parameters were kept the same and stationary laminar flow and solid mechanics equations were solved. Deformation profile, shear stress and pressure were assessed for representative computed settling velocities (${u}_{cell}\sim \left[0.1-1\right] \mathrm{m}/\mathrm{s}$). Effective refractive index variation in time due to a single cell flowing through the Fabry-Pérot cavity was simulated using a custom finite element numerical model of nested ellipsoids, as presented in previously published work^12^. While volumes of each ellipsoid remained constant, cell elongation and nucleus offset in the flowing direction were varied. Specifically, the effective refractive index simulation model is as follows. Spherical radius of the cell membrane and the nucleus were set to 6 μm and 5.45 μm respectively, corresponding to a realistic 0.75 volume ratio. RI of the cytoplasm and nucleus were set to 1.40 and 1.37, respectively. The surrounding RI was set to the one of phosphate-buffered saline (PBS) (n = 1.335). The intensity profile of the optical mode inside of the cavity was modelled as a two-dimensional Gaussian of 20 μm by 7.5 μm (at 3σ). The distance between the distributed Bragg reflectors (the width of the microchannel) was set to 35 μm. For each step of the cell flowing through the cavity, the volume overlap between the cell and the cavity mode intensity was numerically calculated and then projected onto the LP_01_ optical mode of a SMF-28 optical fibre (diameter of 8.2 m) modelled using Bessel functions. The effective RI was calculated as the sum of the projected RIs weighted by their respective area ratios on the LP_01_ mode. Finally, the variation of the calculated RI minus the one of PBS was plotted against time.

### Statistics

Data were tested for significance using paired t test. Numbers of independent repeats are indicated in the figure legends. The minimal significance threshold was set at 0.05 for all tests. *P* values are as follows: ns, non-significant; * *P* value < 0.11 (non-significant, but showing a trend), ** *P* value < 0.05 (significant).

## Results

### Computed operating parameters of interferometric deformability cytometry and curve parameters are related to cellular features

**Figure 1:**
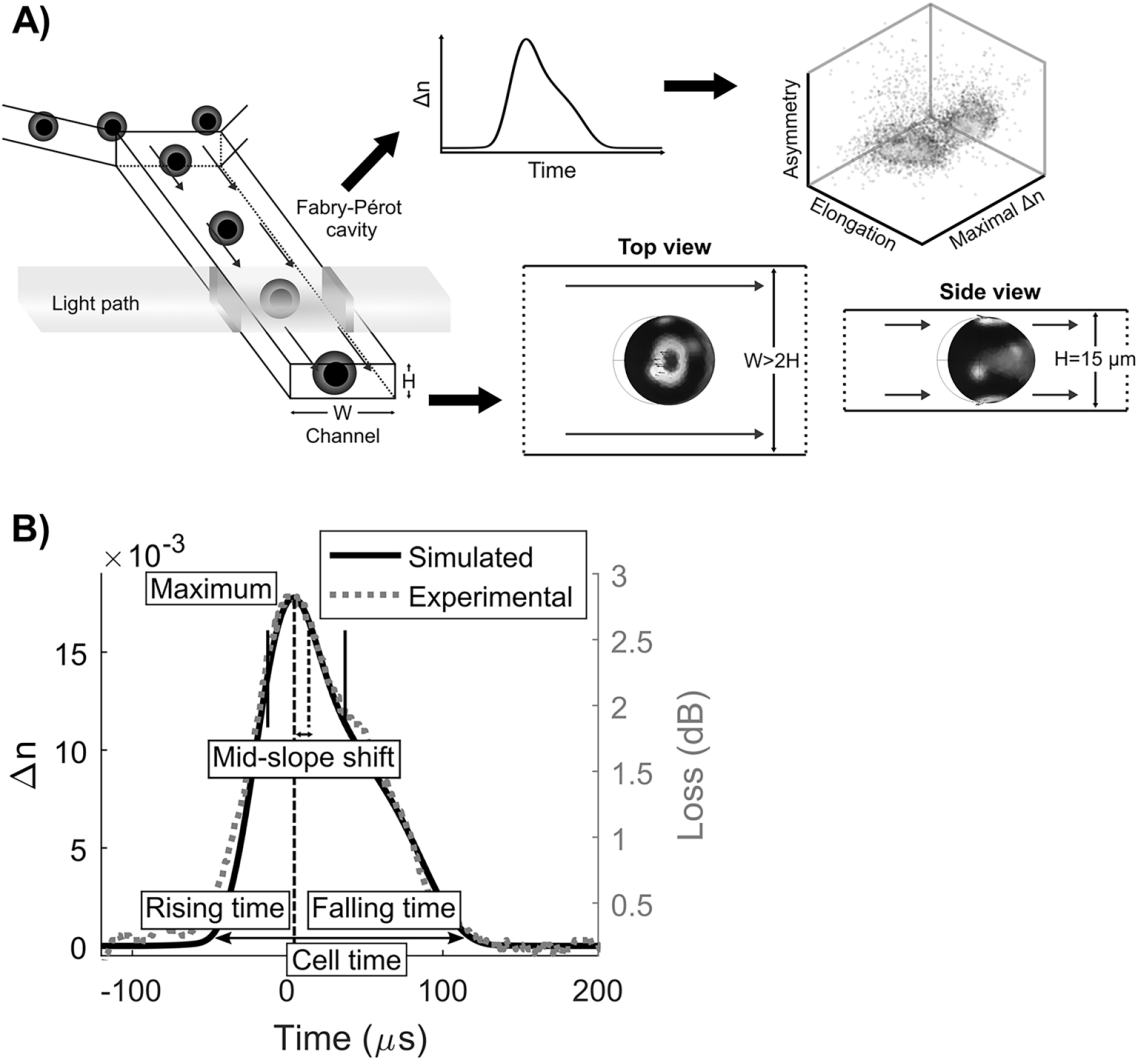
Use of interferometric deformability cytometry to measure mechanical properties of BMDCs. (**A**) Schematic representation of interferometric deformability cytometry showing focused cells, light path, Fabry–Pérot resonant cavity, and deformation profiles of a homogeneous sphere. (**B**) Simulated refractive index variation (Δn) in time overlapped with a typical experimental loss curve of a deformed cell flowing through the sensor cavity. Calculated curve parameters are represented.

Typical shear-induced deformability cytometry devices use a square channel in which symmetrical orthogonal deformation of cells are reported^17^. However, using a small aspect ratio rectangular channel, significant deformation only occurs in the height profile since shear stresses are applied to cell parts closest to the walls (Fig. 1A). Simulation results for a homogeneous sphere corroborate this deformation profile and expose the location of shear stresses and pressure (Fig. S1). For a simulated settling cell velocity range of ${u}_{cell}\sim \left[0.1-1\right] \mathrm{m}/\mathrm{s}$, the corresponding computed shear stress, mean strain, stimulation time, and strain rate are in the order of $\sigma \sim \left[0.1-1\right] \mathrm{kPa}$, $\underset{\_}{\varepsilon }\sim \left[10-50\right]\%$, $\Delta t\sim \left[10-1\right] \mathrm{ms}$ and $\dot{\varepsilon }\sim \left[0.01-0.5\right] \mathrm{kHz}$ respectively. These values are in agreement with previous published works on shear-induced deformation^13^. Within these parameters, we observed typical curves of simulated refractive index variation ($\Delta n$) and experimentally measured loss in time (Fig. 1B). The validity of our effective refractive index numerical model was supported by the apparent similarities between simulated effective refractive index change and experimentally measured loss profiles. Systematic variations of the nested ellipsoids geometries allow to correlate cellular properties to curve parameters (Fig. S2).

From the many explored curve parameters, we only report here the ones having a direct and clear relationship to a cellular feature, that is *maximum*, *cell time*,* time ratio* and the *mid-slope shift* (Table 1). As represented in Fig. 1B and Table 1, *maximum* is the maximal point reached by the curve and thus represents the highest RI variation measured. This parameter was found to be most sensitive to the cytoplasmic volume. *Cell time* is the time span of the cell in the cavity representing the length of the cell and defines whole-cell elongation. *Time ratio* is the ratio of *rising time* (starting point of curve to *maximum* position) over *falling time* (*maximum* position to ending point of curve) and represents the asymmetry of the curve. *Mid-slope shift* is the difference between the center of the maximal slopes and the *maximum* position normalized by the *cell time*. *Time ratio* and *mid-slope shift* both relate to the RI spatial distribution for which the nucleus offset has the greatest influence. Notably, in our simulations, the *mid-slope shift* parameter only reported variations if the nucleus had a smaller RI value than the cytoplasm. This is in line with other studies reporting smaller RI values for the nucleus than the cytoplasm^18–20^. As immature and mature BMDCs are known to present with distinct physical properties, we aim to characterize their respective intracellular organization patterns by assessing these 4 different parameters using our interferometric deformability cytometry method.

**Table 1:** Relationships between calculated parameters and cellular features.

Parameter name	Curve attribute	Cellular feature
*Maximum*	Curve maximum	Cytoplasmic volume
*Cell time*	Curve time span	Whole-cell elongation
*Time ratio*	Rising time/falling time	Spatial RI distribution asymmetry
*Mid-slope shift*	(Maximal slopes center-maximum)/cell time	Nucleus shift from cell center

### Immature BMDCs nucleus position depends on fluidic stress amplitude

**Figure 2:**
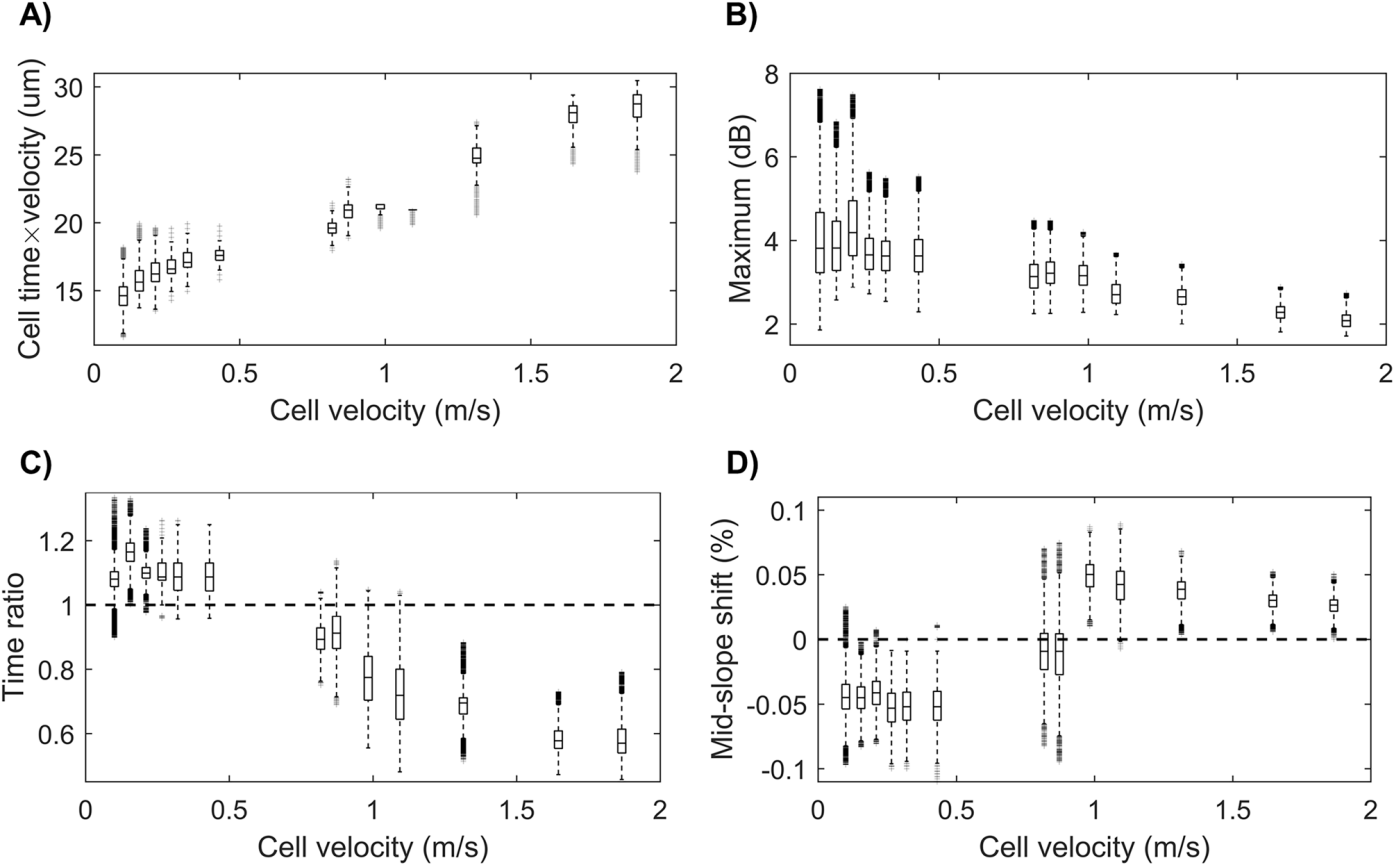
Interferometric deformability cytometry properties of immature BMDCs. (**A**) Cell length calculated as *cell time* × velocity, (**B**) *maximum*, (**C**) *time ratio* and (**D**) *mid-slope shift*, represented at varying cell velocities (x axis, m/s). Horizontal dashed lines are the respective parameter values of a centered nucleus with a *time ratio* at 1 and a *mid-slope shift* at 0. Data was acquired in three independent experiments, where each experiment tested different velocities. Experiment 1 acquired data for the three lowest cell velocities, experiment 2 for the five subsequent cell velocities and experiment 3 for the 5 highest cell velocities. Each cell velocity was acquired on various numbers of cells ranging from 13,292 to 28,437 cells, with an average of ~ 22,000 cells per velocity. For each velocity, a box plot is used to represent the measured parameter population*.* The box plots show the median value, upper and lower quartile (box), maximum and minimum values for each sample.

Immature BMDCs were prepared from bone marrow cultures in the presence of FLT3L and analyzed by flow cytometry. The cell preparations were relatively homogeneous and the immature phenotype was confirmed by the low expression of MHC II (I-A), CD40, CD80 and CD86 (Fig. S3). The immature BMDCs were then analyzed by interferometric deformability cytometry. As for flow cytometry, debris and dead cells are excluded for the analysis (Fig. S4). All of the parameters measured by interferometric deformability cytometry on immature BMDCs show that immature BMDCs are relatively homogeneous (Fig. 2), thus confirming the optomechanically similar phenotype of these cells. For the largest flow reported, the time required to perform a single cell measurement was 25 µs, thus corresponding to a maximal rate of 40,000 cells/s. To determine how fluidic stress amplitude influences the shape of immature BMDCs, velocities were adjusted to approximately match the size of an undeformed cell at the smallest flow, and increased thereafter. As expected, *cell length* (*cell time* × velocity) increases with increasing cell velocity (Fig. 2A), suggesting that the immature BMDCs are stretched as the fluidic stress increases. Conversely, *maximum* decreases with increasing cell velocity (Fig. 2B). This suggests that as fluidic stress amplitude increases with cell velocity, cell elongation also tends to increase. Accordingly, less cell volume is interacting with the optical Gaussian mode effective width, hence the decrease in *maximum*. For this parameter, the population spread also decreases with increasing cell velocity. The combination of stronger inertial focusing forces at larger velocities leading to a complete focusing of the cells and a smaller spread in cell volume interacting with the effective optical width due to deformation can explain this phenomenon. Interestingly, *time ratio* and *mid-slope shift* trends differ from their intuitive behavior. Indeed, at smaller velocities both parameters suggest a nucleus located towards the front-end of the cell, as reported by the $>1$ *time ratio* and $<0$ *mid-slope shift* parameters (Fig. 2C,D), rather than being centered, as one would expect. As cell velocity increases, *time ratio* becomes smaller than one and *mid-slope shift* becomes larger than zero, indicating a nucleus passing the cell center and moving towards the trailing-end of the cell. These unexpected results suggest a repositioning of the nucleus in function of fluidic stress amplitude. Noticeably, two preferred intracellular nucleus positions, front-end and back-end, are observed, based on small or large fluidic stresses, respectively. For this device, the inflexion point is between 0.5 and 1 m/s in cell velocity, creating a clear divide between small and large fluidic stress based on a *time ratio* threshold of 1 and a *mid-slope shift* threshold of 0. Hereafter, small fluidic stress refers to *time ratio* > 1, whereas large fluidic stress refers to *time ratio* < 1.

### Mature BMDCs are stiffer and display lower nucleus mobility

Since fluidic stress amplitude induces different nuclear positioning in immature BMDCs, it is reasonable to believe that intracellular mechanical phenotyping can provide biophysical insights on the maturation process of BMDCs. Indeed, maturation of BMDCs is expected to produce measurable optomechanical changes, especially in terms of cytoplasmic content, cellular stiffness, and nucleus mobility. Thus, we decided to compare interferometric deformability cytometry parameters between immature and mature BMDCs at small and large fluidic stress regimes. As such, we produced immature and mature BMDCs in culture and, prior to analysis by interferometric deformability cytometry, their phenotypes were confirmed by flow cytometry. Specifically, mature BMDCs expressed higher levels of MHC II, CD40, CD80 and CD86 relative to immature BMDCs (Fig. S3). Importantly, immature and mature BMDCs show overlapping forward scatter profiles (Fig. S3), demonstrating that LPS treatment does not significantly alter cell size.

**Figure 3:**
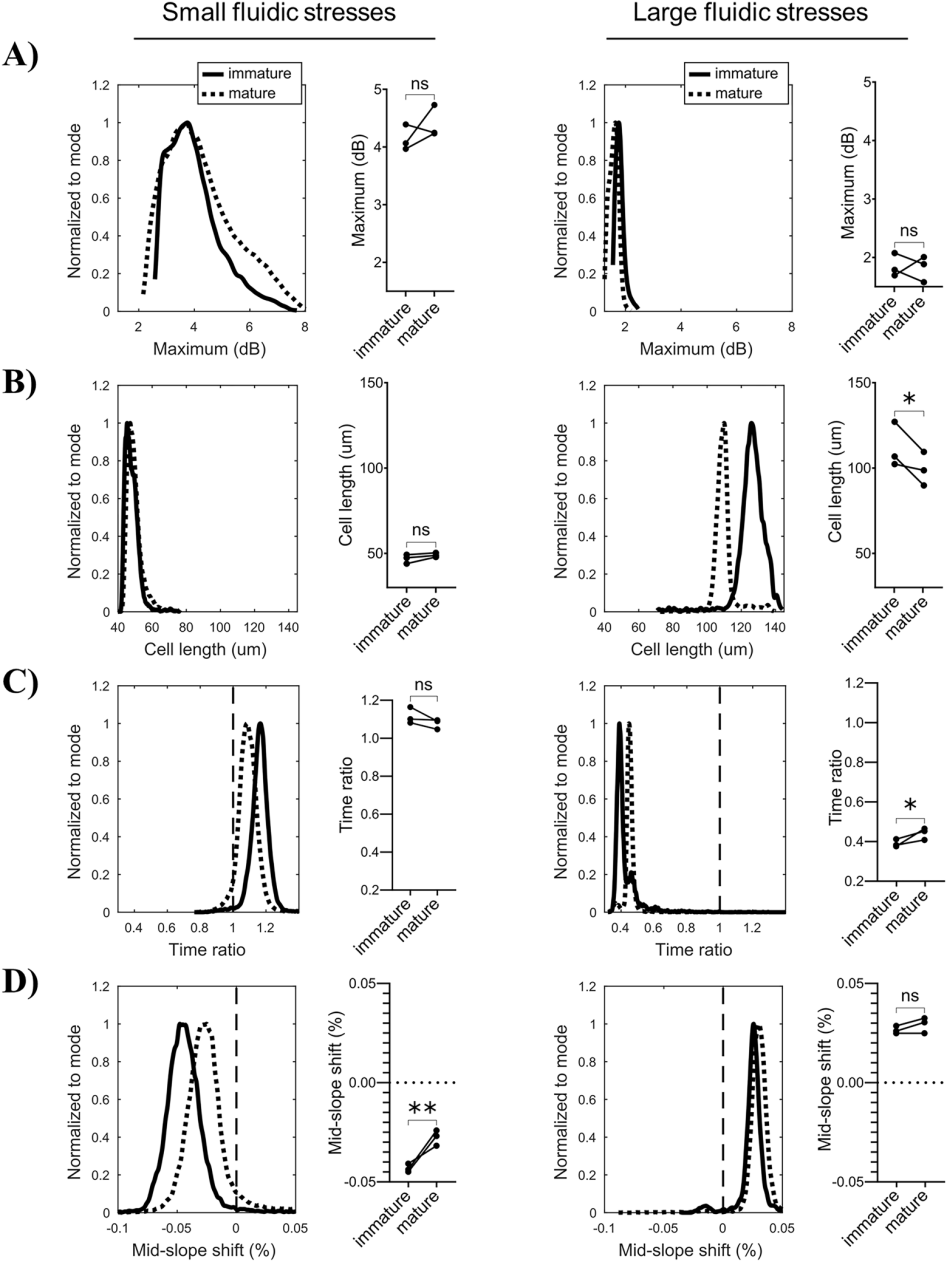
Comparison of physical parameters between immature BMDCs and mature BMDCs. Both immature and mature BMDCs were subjected to (left) small and (right) large fluidic stresses, (Fig. 2), respectively, and cellular physical parameters were quantified by interferometric deformability cytometry. Triplicate linked means are plotted on the right side and representative histograms are illustrated on the left, for *maximum* (**A**), *cell length* (**B**), *time ratio* (**C**) and *mid-slope shift* (**D**). The data was acquired in three independent experiments. Statistical significance was calculated using paired t test. *P* values are as follows: *ns* non-significant; *P value < 0.11 (non-significant, but showing a trend), **P value < 0.05 (significant). Vertical lines indicated a centered nucleus.

Immature and mature BMDCs were first subjected to small fluidic stresses and cellular parameters were quantified by interferometric deformability cytometry. At small fluidic stresses (corresponding to ~ less 0.75 m/s in this device, Fig. 2), there is no statistical difference on the *maximum* and *cell length* parameters between immature (solid line) and mature (dotted line) BMDCs (Fig. 3A,B). This was expected since small stresses should not produce significant cellular deformation, and both immature and mature BMDCs are of similar size (Fig. S3). *Time ratio* and *mid-slope shift* parameters both report more symmetrical RI spatial distribution for mature BMDCs, although only the *mid-slope shift* reports a statistically significant difference, when compared to immature BMDCs (Fig. 3C,D). This corresponds to a smaller nucleus offset in mature BMDCs relative to immature BMDCs. Notably, each point represents data acquired at different cell velocities below 0.75 m/s, namely at 0.3, 0.47, 0.63 m/s, and are paired for immature and mature BMDCs in each experiment. The high reproducibility of the data at different cell velocities clearly indicates that both immature and mature BMDCs have a specific behavior in a large range of small fluidic stress, with a front-end nucleus position, in line with the data presented in Fig. 2.

Similarly, we applied two different large fluidic stresses, with cell velocities of 0.86, 0.86 and 1.15 m/s, to both immature and mature BMDCs. At these large fluidic stresses, the *maximum* of both immature and mature BDMCs reduces dramatically when compared to small fluidic stresses (Fig. 3A). However, at these large fluidic stresses, there tends to be a difference between immature and mature BMDCs on *cell length,* with mature BMDCs showing shorter length (Fig. 3B). This suggests that mature BMDCs are stiffer and do not elongate as much as immature BMDCs when subjected to comparable fluidic stress. In addition, at large fluidic stress, mature BMDCs tend to show an increase in *time ratio* relative to immature BMDCs, while *mid-slope shift* is comparable for both immature and mature BMDCs (Fig. 3C,D). The slight increase in *time ratio* for mature BMDCs suggests a smaller nucleus offset than for immature BMDCs. Altogether, these results suggest that mature BMDCs are stiffer and display less nucleus mobility than immature DCs. Moreover, we find that *mid-slope shift* is a better indicator of nucleus offset at small stresses whereas *time ratio* is more adapted for large stresses.

### Immature and mature BMDCs can be discriminated using interferometric deformability cytometry

**Figure 4:**
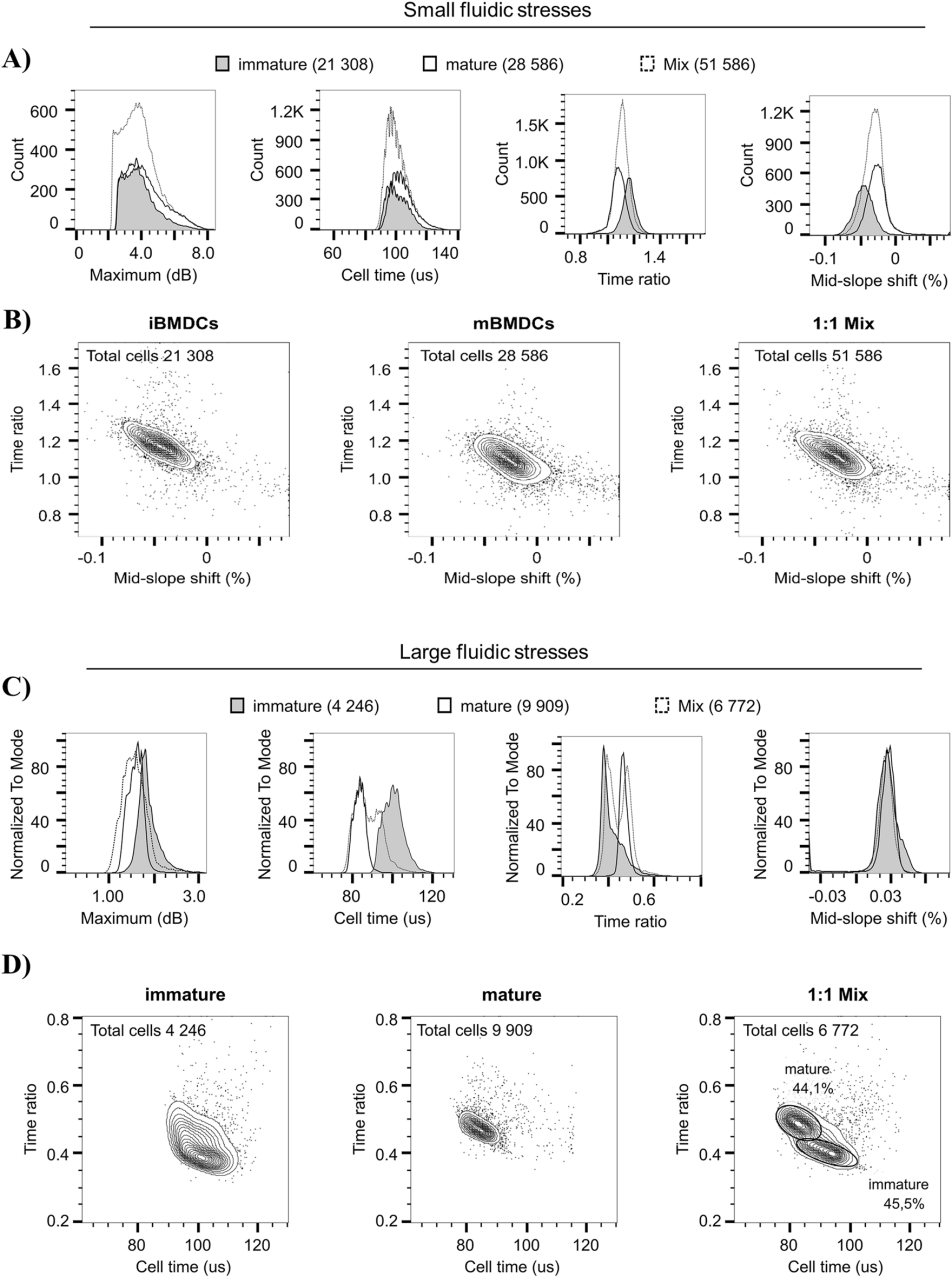
Interferometric deformability cytometry discriminates between immature BMDCs and mature BMDCs. The histograms represent the overlay of the *maximum*, *cell time*, *time ratio* and *mid-slope shift* parameters for immature BMDCs, mature BMDCs or a 1:1 mixed cell suspension at small (**A**) or large (**C**) fluidic stress. Contour plot of gated immature BMDCs, mature BMDCs and a 1:1 mixed cell suspension in function of *mid-slope shift* vs *time ratio* (**B**) or *cell time* vs *time ratio* (**D**) measured at small (**B**) or large (**D**) fluidic stresses. Small stress was achieved using a 50 µm × 15 µm channel (AR = 0.3) at Q = 15 µl/min corresponding to a computed maximal fluid velocity of 0.6 m/s and a cell velocity of 0.47 m/s (**A,B**) and larger stress, using a 30 µm × 15 µm channel (AR = 0.5) at Q = 20 µl/min corresponding to a computed maximal fluid velocity of 1.5 m/s and a cell velocity of 1.15 m/s (**C,D**). The duration of a single cell measurement was 190 µs, thus corresponding to a maximal rate of approximately 5000 cells/s. One representative of two experiments depicts the qualitative separation of immature and mature BMDCs using interferometric deformability cytometry.

Characterizing the optomechanical phenotype of separate BMDCs populations provides important knowledge on their biophysical behavior. The most common tool used to characterize DC properties is, arguably, flow cytometry. However, flow cytometry does not allow to distinguish immature and mature BMDCs based on their physical properties alone, and surface protein expression must be assessed by using fluorescence-coupled antibodies. Indeed, although granularity is slightly increased in mature relative to immature BMDCs, there is not a full shift in the cell population and there remains a significant overlap (Fig. S3). As such, these immature and mature cell states do not substantially differ in cell size and granularity (Fig. S3), the two physical parameters quantified by flow cytometry. Having observed that various parameters differ between immature and mature BMDCs when analyzed by interferometric deformability cytometry (Fig. 3), we aimed to determine whether interferometric deformability cytometry could be used to discriminate immature and mature BMDCs. We performed a side-by-side comparison of immature and mature BMDCs, as well as a sample where both cell subsets were mixed in a 1:1 ratio. The mixture was then subjected to small and large fluidic stresses. At small stresses, as expected from our analysis on separate populations (Fig. 3), *maximum* and *cell time* profiles overlapped for immature and mature BMDCs, and thus also for the mixed sample (Fig. 4A). Correspondingly, *time ratio* and *mid-slope shift* were respectively lower and higher for mature BMDCs relative to immature BMDCs (Fig. 4A), again supporting a shift in the position of the nucleus based on maturation status of the BMDCs. Still, these shifts in distribution are relatively minor, such that by plotting these two parameters in a contour plot, it was impossible to distinguish the immature and mature BMDCs in the mixed sample (Fig. 4B). At large fluidic stresses, we again find that *cell time* of mature BMDCs is lower than that of immature BMDCs, while *time ratio* of mature BMDCs is higher than that of immature BMDCs (Fig. 4C). By plotting the data as *time ratio* versus *cell time*, we can observe a distinction between immature and mature BMDCs (Fig. 4D). Indeed, the sample containing a 1:1 mixture of immature and mature BMDCs qualitatively resolves in two distinct subsets (Fig. 4D). Of note, *maximum*, *time ratio* and *mid-slope shift* parameters assessed on the mixed population yield similar profiles to the ones from the individual BMDC preparations (Fig. 4C). However, immature BMDCs present with a higher *cell time* value, i.e. elongation, in comparison to the mix population (Fig. 4C). Overall, this proof of concept presents evidence that our interferometric deformability cytometry method allows to discriminate immature and mature BMDCs uniquely based on their deformability and intracellular optomechanical properties.

## Discussion

By using our interferometric deformability cytometry technology^12^, we have been able to discriminate between two states of DC activation, immature versus mature. The discrimination is based on a combination of quantifiable parameters that reflect differences in nuclei position, stiffness, and their ability to deform under physical stress. Ex vivo derived DCs are of interest in the next generation of DC based vaccines, notably for anti-cancer therapeutic^21^. However, to date, various challenges limit their effectiveness in treatments. For instance, DCs need to be mature and able to migrate to the tumour^22^. Despite a lot of interest for the role of chemokines in the migration of DCs^3^, few studies have explored their mechanical properties, even if evidence suggests that they are closely linked to their migration potential and function^5, 23^.

Although simplistic, our simulation model replicating RI variation in time can almost perfectly predict the experimentally recorded signal and helped define the relationships between curve parameters and cellular features. The remaining divergences observed between simulated and experimental signals are fluctuations before and after the main curve as well as rising and falling slopes mismatch. Fluctuations are believed to be due to fluidic pressure effects on the reflectors. Indeed, the small thickness of the distributed Bragg reflectors silicon layers on the microchannel end (between 1 and 2 μm) confers some mechanical flexibility^24^. As for the slopes mismatch, non-elliptic profiles of the deformed cells and protein vesicles of large RI are presumed to be responsible for this discrepancy. Indeed, deformation profiles generated by finite element method simulations were asymmetrical with respect to the polar axis. However, such simulation can only give a general idea of the fluidic stresses, deformation profile and settling velocity. Approximating an entity as complex as the cell by a homogeneous sphere is a valid method, yet is far from the reality and must be tested experimentally.

Contemporary studies exploiting deformability cytometry suggest that deeper structures, such as the nucleus, are only involved when large stresses and strain rates are exerted^13^. Our results show that intracellular organization occurs even at very small stresses, as reported by the front-leading position of the nucleus at small velocities. This behavior is in striking contrast to the assumption that a nucleus should generally be centered when no significant deformation occurs. Moreover, it suggests the existence of an external forces transmission mechanism. However, exact biomechanical entities involved in such nucleus ordering and mobility at small stresses remain unknown. Further experiments are necessary to elucidate this complex behavior of the cell. In addition to simulations and deformability cytometry, other methods should be used to determine the specific influence and impact of nucleus position, cellular stiffness, and deformation on the discrimination between immature and mature DCs.

Mature BMDCs deform less, and are thus stiffer, than immature BMDCs. Indeed, cellular stiffening following maturation for BMDCs has been quantified using atomic force microscopy, as well as for DCs using parallel plates rheology or dielectrophoresis stretching^4, 8, 23^. Together these studies show that DC stiffening upon maturation correlates with a larger actin content and an increased actin functionality. This stiffening is in line with a recent RT-deformability cytometry study showing similar behavior of mature moDCs due to an increased actin cortex network^7^. Mature BMDCs have a reduced nuclear mobility relative to immature BMDCs. It is reasonable to believe that the perinuclear actin network described in immature BMDCs^9^ might be even more developed in mature BMDCs. Overall, as the main role of mature DCs is to interact with naive T cells long enough to induce their activation, it may require that the DCs be stiffer to stabilize this interaction. In contrast, immature BMDCs must extend their dendrites to screen the environment to capture the antigen. This feature may require a cell that is more flexible, thus less stiff. Our study adds to previous work by showing that the distinct physical properties of immature and mature BMDCs can be distinguished using interferometric deformability cytometry at cell rate comparable to that of current state-of-the-art flow cytometers.

The similar values of the *maximum* parameter for immature and mature BMDCs suggest that the nucleus volume was not altered by the LPS stimulation. Indeed, in our assay, this parameter was most sensitive to the cytoplasmic volume, which in this case is indicative of the nucleus size since the cell size did not vary. Interestingly, the two parameters describing the nucleus position, *time ratio* and *mid-slope shift*, are useful in different conditions. *Mid-slope shift* yields better separation than *time ratio* at small fluidic stresses whereas it is the opposite at larger fluidic stresses. When little elongation occurs, the cell occupies more volume at the peak of the Gaussian optical mode. This greater light-cell interaction yields better cellular RI sensing which translates to larger loss differences on the curve. Since *mid-slope shift* is computed from the maximal slopes of the curve, this parameter is better suited for small cellular deformation. In contrast, *time ratio* computes the starting, ending, and maximal points of the curve which are exaggerated and easily identified on deformed cells. Still, discrimination of immature and mature BMDCs in a mixed sample was not possible using the *mid-slope shift* parameter at small fluidic stresses whereas *cell time* and *time ratio* yielded clear discrimination at larger fluidic stresses. Interestingly, when mixed with mature BMDCs, *cell time* of immature BMDCs slightly shifted towards the values of mature cells. One hypothesis is that mature BMDCs interact with immature BMDCs when mixed together, which in turn might slightly decrease deformability. Additional experiments are required to define the cause of this shift in cell time. Finally, in our setting, *mid-slope shift* is a better indicator of nucleus offset at small stresses whereas *time ratio* is more adapted for large stresses.

In conclusion, using a Fabry-Pérot as a sensitive intracellular probing method has revealed unexpected mechanics of BMDCs. Although the extensive effect of the nucleus on whole-cell deformability was already established^13^, single cell characterization of the nucleus position and mobility had not been demonstrated. Furthermore, we have shown that cellular deformation is not the only mechanical feature capable to assess phenotypic differences upon DC maturation. Overall, interferometric deformability cytometry improves intracellular mechanical phenotyping of single cells at a high throughput, opening the way for more precise characterization of large populations. This, in turn, will contribute to the advancement of novel technologies that will increase our knowledge of DC mechanic properties. As DCs are a highly heterogeneous population, further studies of different ex vivo isolated DC subsets may allow to determine their migration and functional abilities.

## Supplementary Information

Supplementary Figures.

## Data Availability

The datasets generated and analyzed during the current study are not publicly available due to the absence of public databases allowing for sharing of raw flow cytometry data but are available from the corresponding author on reasonable request.
