# Supplementary material for: Immature and mature bone marrow-derived dendritic cells exhibit distinct intracellular mechanical properties
Source: Sci Rep. 2023 Feb 3;13:1967. doi: 10.1038/s41598-023-28625-w (PMC9898242; doi:10.1038/s41598-023-28625-w)
Supplement: Supplementary file 1 — Supplementary Figures. [file 41598_2023_28625_MOESM1_ESM.pdf]

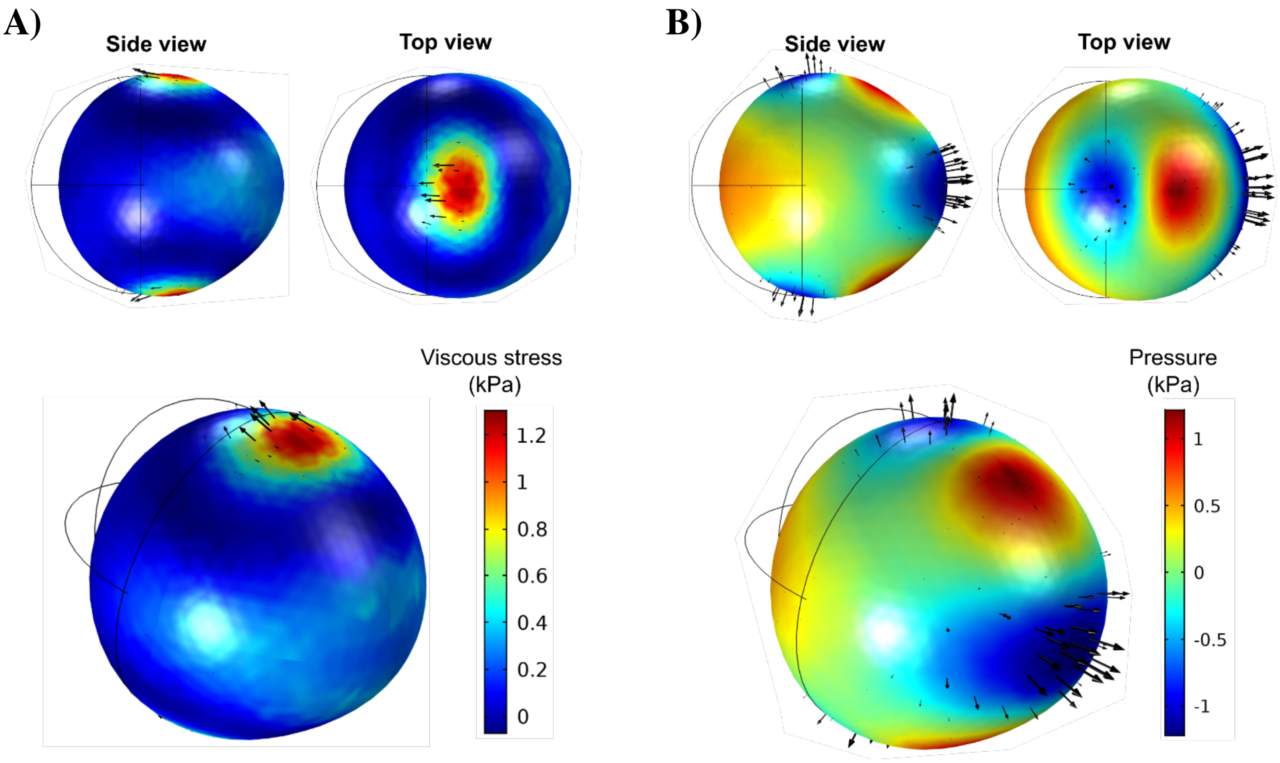

Figure S1: Finite element method simulation results showing the deformation profiles of a homogenous sphere as well as the location and amplitude of shear stress (A) and pressure (B).

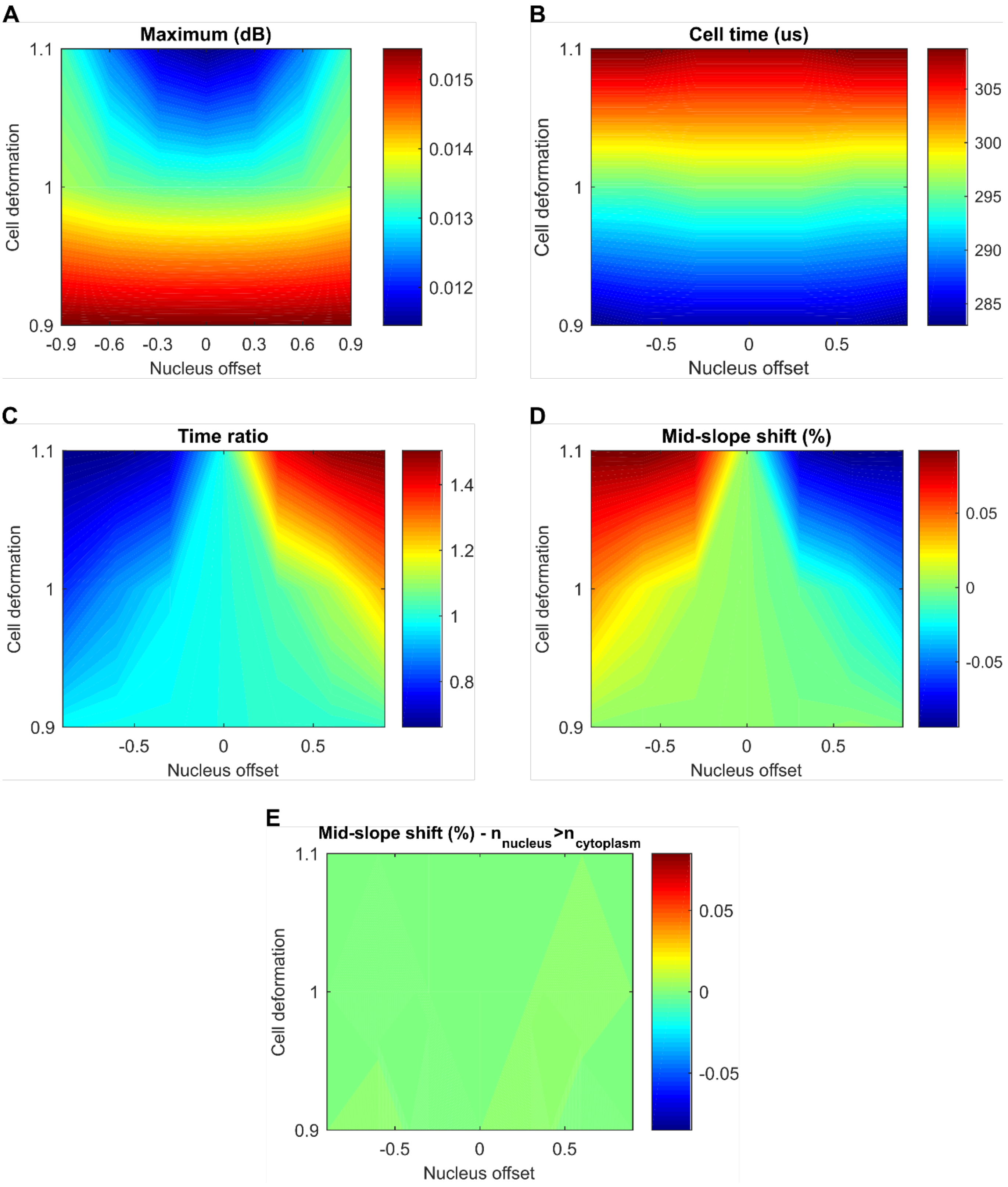

Figure S2: Variation of A) *maximum*, B) *cell time*, C) *time ratio* and D) *mid-slope shift* in function of nucleus shift and cell deformation. E) A nucleus having a larger refractive index than the cytoplasm showing no variation in mid-slope shift.

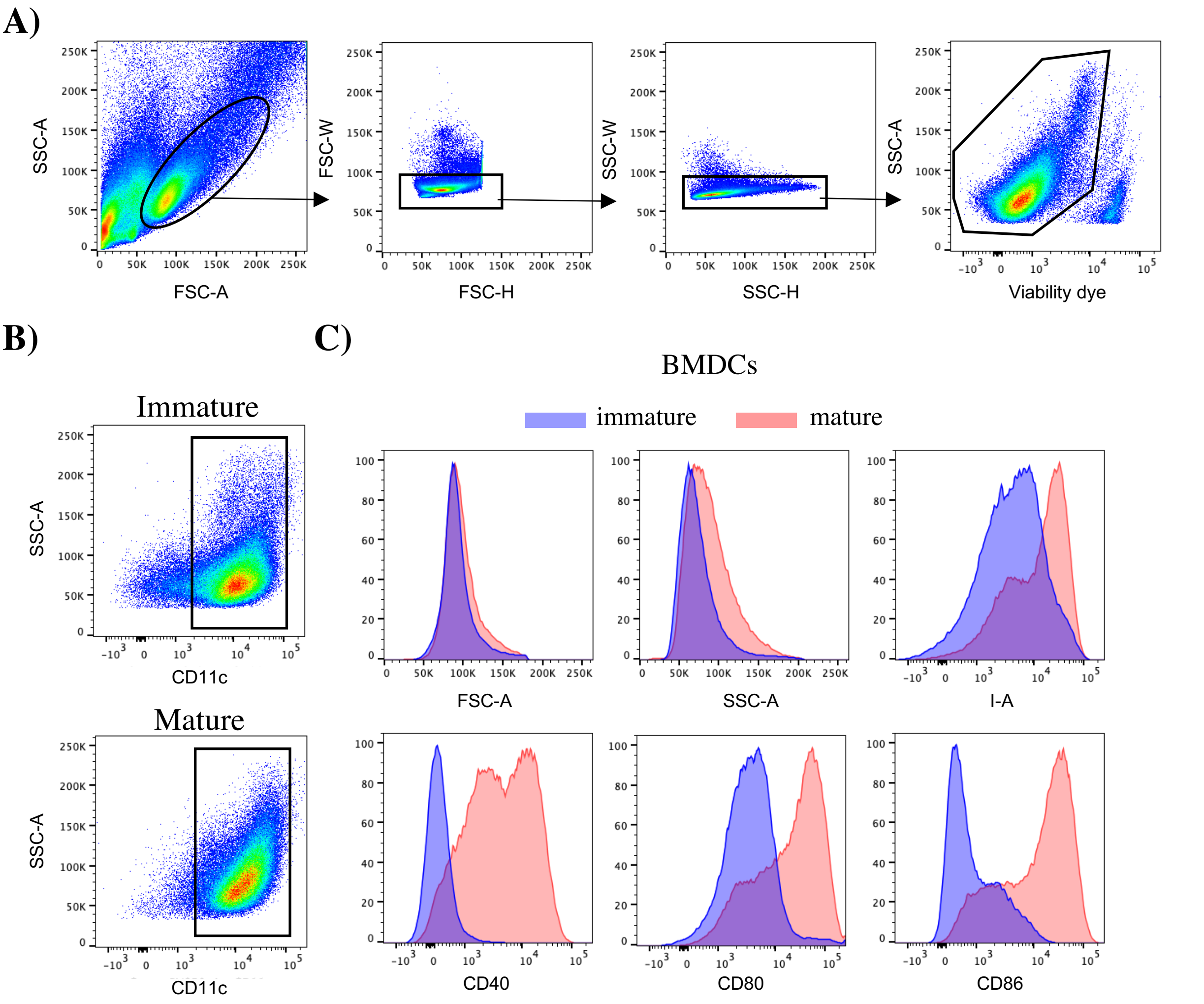

Figure S3: Representative gating strategy and phenotype for immature and mature BDMCs. (A) Exclusion of debris, doublets and dead cells, to select for (B) immature and mature BMDCs based on the expression of CD11c. (C) Overlay of various properties of $CD11c^{+}$ immature (purple) and mature (pink) BMDCs detected by flow cytometry, namely forward scatter (FSC) and side scatter (SSC), as well as the expression levels of proteins increased with BMDC maturations MHC-II (I-A), CD40, CD80, CD86.

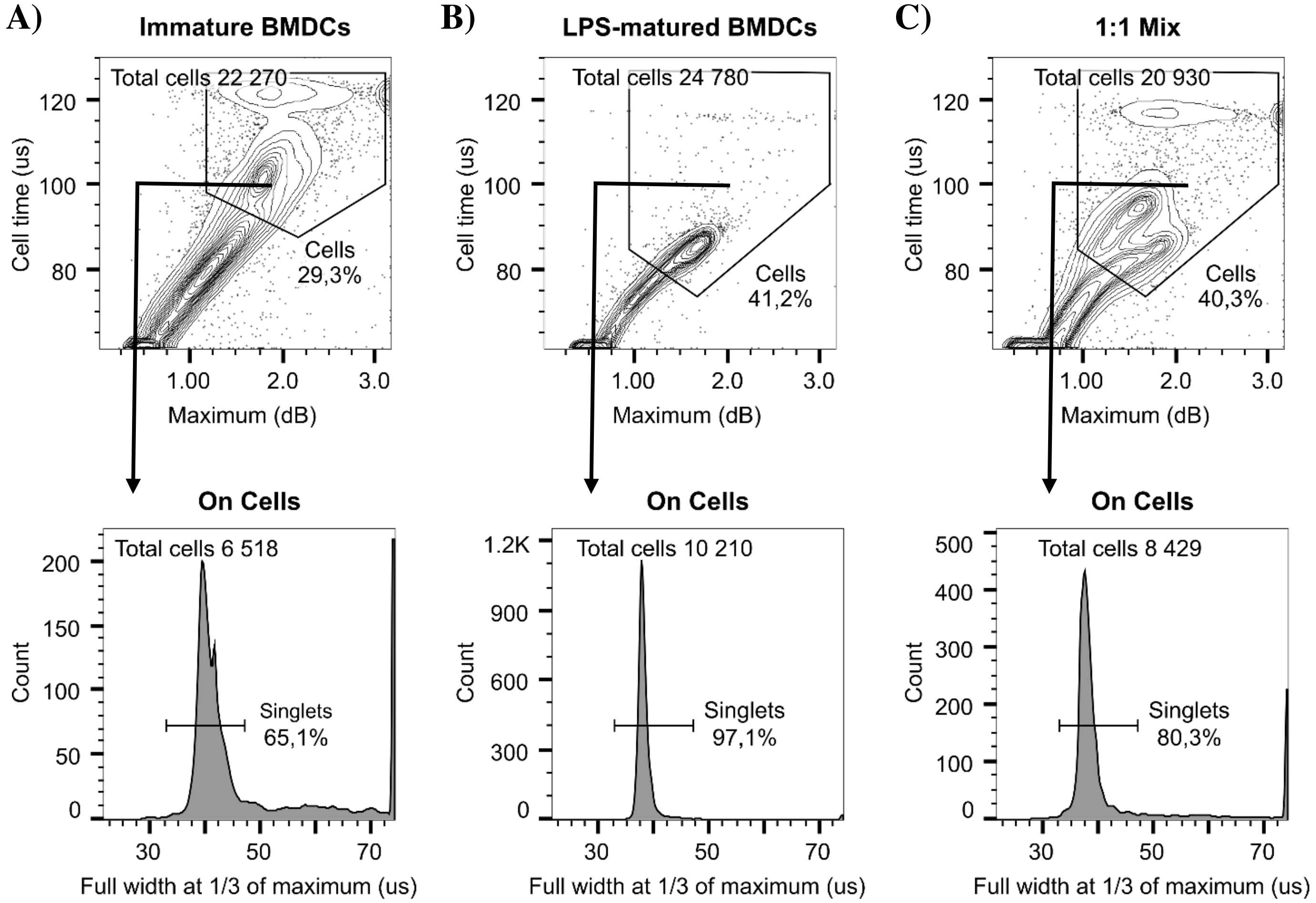

Figure S4: Interferometric deformability cytometry exclusion gating strategy employed for all analyses. First, debris and dead cells are excluded based on their smaller *maximum* and *cell ti*me values. Then, doublets and artefacts are excluded based on their larger curve width, using the Full width at 1/3 of the maximum (FWTM). This experiment was realized using a 30 µm×15 µm channel (AR=0.5) at Q = 20 µl/min corresponding to a computed maximal fluid velocity of 1.5 m/s and a cell velocity of 1.15 m/s. One representative of three experiments for A) immature BMDCs, B) mature BMDCs and C) a 1:1 ratio mix of immature and mature BMDCs.
